# Asthma and the risk of lung cancer: a meta-analysis

**DOI:** 10.18632/oncotarget.14595

**Published:** 2017-01-11

**Authors:** Yan-Liang Qu, Jun Liu, Li-Xin Zhang, Chun-Min Wu, Ai-Jie Chu, Bao-Lei Wen, Chao Ma, Xu-yan Yan, Xin Zhang, De-Ming Wang, Xin Lv, Shu-Jian Hou

**Affiliations:** ^1^ Department of Anesthesiology, Hospital of PLA, Qingdao 266071, Shandong, China; ^2^ Department of Anesthesiology, Shanghai Pulmonary Hospital, Tongji University School of Medicine, Shanghai, China; ^3^ Department of Hand Surgery, Hospital of PLA, Qingdao 266071, Shandong, China

**Keywords:** asthma, lung cancer, association, meta-analysis

## Abstract

Some studies found that there was a significant association between asthma and the risk of lung cancer. However, the results are inconclusive. Therefore, we performed a meta-analysis. We searched the electronic databases for all relevant articles. Odds ratio (OR) with 95% confidence interval (CI) were used to calculate the strength of the association between asthma and lung cancer risk. Asthma was significantly associated with the increased risk of lung cancer (OR = 1.44; 95% CI 1.31–1.59; *P* < 0.00001; *I^2^* = 83%). Additionally, asthma patients without smoking also had the increased lung cancer risk. In the subgroup analysis of race and gender, Caucasians, Asians, male, and female patients with asthma showed the increased risk of lung cancer. However, asthma was not significantly associated with lung adenocarcinoma risk. In the stratified analysis by asthma definition, significant associations were found between asthma and lung cancer in self-reported subgroup, questionnaire subgroup, and register databases subgroup. However, no significant association was observed in physician-diagnosed asthma subgroup. In conclusion, this meta-analysis suggested that asthma might be significantly associated with lung cancer risk.

## INTRODUCTION

Lung cancer is one of the most common malignant tumors affecting millions of people around the world. It is acknowledged that smoking is the most important risk factor of lung cancer [1]. Sun et al. suggested that approximately 25% of lung cancer cases worldwide are not attributable to tobacco use [2]. This fact indicated that other factors might contribute to susceptibility to lung cancer.

Asthma is one of the most common diseases of childhood, with an estimated global prevalence of 10% among children aged 6–7 [3]. It is a condition characterized by chronic inflammation of the lungs, presenting with airway hyper-reactivity, excessive mucous formation, and respiratory obstruction. The chronic inflammation had an important role in the cancer development [4]. Thus, the chronic inflammation in conditions such as asthma might lead to lung cancer development. Some studies found that there was a significant association between asthma and the risk of lung cancer. However, the results are inconclusive [5–22]. Therefore, we performed a meta-analysis to determine the association between asthma and lung cancer risk.

## RESULTS

### Characteristics of eligible studies

**Figure 1:**
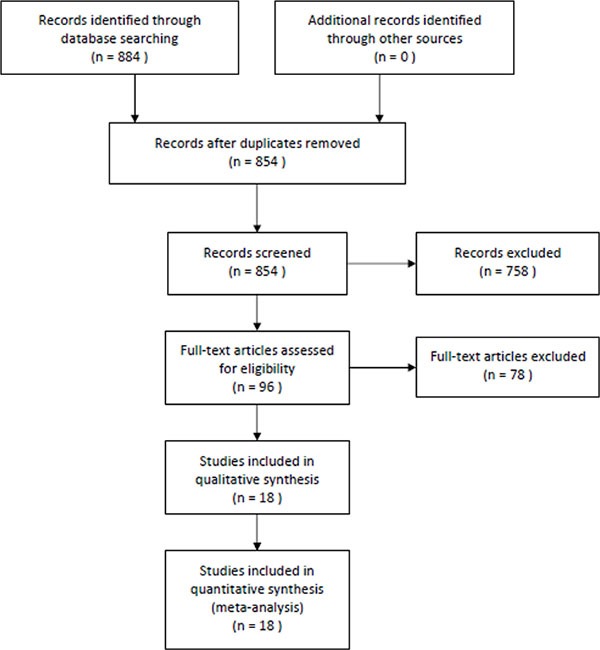
The selection of included studies

The detailed literature search strategy was showed in Figure 1. A total of 884 potential studies were identified by preliminary searching. After carefully review, 18 studies were included in this meta-analysis [5–22]. Four studies reported two independent studies. Thus, 22 studies with 16375202 subjects were included. Table 1 presents the main characteristics of these studies, such as first author's name, year of publication, ethnicity, gender, age, asthma definition, lung cancer definition, duration of follow-up, sample size, and adjustment. The quality of the studies was high.

**Table 1:** Characteristics of the included studies

First author	Year	Race	Gender	Age (y)	Definition of asthma	Definition of lung cancer	Follow-up years	Samle size	Covariants	NOS
Alderson	1974	Mixed	Mixed	25–59	Self-reported	Obtain from register databases	21	1892	No	7
Reynolds 1	1987	Mixed	Male	NA	Self-reported	Obtain from register databases	18	3117	Age, smoking	9
Reynolds 2	1987	Mixed	Female	NA	Self-reported	Obtain from register databases	18	3708	Age, smoking	9
Mills	1992	Caucasian	Mixed	NA	Questionnaire	Mailed questionnaires and tumor registries	6	34198	Age, sex, smoking history, and time since last physician contact	9
Vesterinen	1993	Caucasian	Mixed	NA	Obtain from register databases	Obtain from register databases	7	35126	No	7
Eriksson	1995	Caucasian	Mixed	16–79	Physician-diagnosed	Obtain from register databases	14	6593	No	7
Huovinen	1997	Caucasian	Male	> 18	Self-reported	Obtain from register databases	16	14654	Age, smoking, social class, pets, dogs, chronic bronchitis, dyspnoea, hay fever	9
Boffetta 1	2002	Caucasian	Male	> 20	Obtain from register databases	Obtain from register databases	8.5	42663	Duration of follow-up, calendar year at entry, age, other diagnoses, emphysema, chronic bronchitis	9
Boffetta 2	2002	Caucasian	Female	> 20	Obtain from register databases	Obtain from register databases	8.5	50323	Duration of follow-up, calendar year at entry, age, other diagnoses, emphysema, chronic bronchitis	9
Talbot-Smith 1	2003	Caucasian	Male	NA	Physician-diagnosed	Obtain from register databases	19	124	Age, smoking	8
Talbot-Smith 2	2003	Caucasian	Female	NA	Physician-diagnosed	Obtain from register databases	19	155	Age, smoking	8
Vandentorren	2003	Mixed	Mixed	25–59	Questionnaire	Obtain from register databases	25	14286	Age, sex, educational level, smoking habit, occupational exposure and forced expiratory volume in one second	9
Littman	2004	Mixed	Mixed	44–74	Self-reported	Obtain from register databases	9.1	17698	Sex, exposure cohort, study arm, education, body mass index, years smoked and years smoked squared, average number of cigarettes smoked per day and average number of cigarettes smoked per day squared, and all other lung diseases, and stratified by smoking status	9
Turner	2005	Mixed	Mixed	> 30	Self-reported	Obtain from register databases	19	26097	Age, gender, race, smoking, education, marital status, body mass index, occupational exposures, beer, wine, and liquor consumption, chronic bronchitis, emphysema, tuberculosis, intakes of vegetables, fruit, fiber, and fat, and passive smoking	9
Brown	2006	Mixed	Mixed	50–89	Self-reported	Self-reported	9	8896	Age, smoking, sociodemographics	8
Gonzalez-Perez	2006	Mixed	Mixed	20–79	Self-reported	Mailed questionnaires	7	18792	Age, sex, calendar year, body mass index, alcohol intake, smoking status, prior comorbidities, health services utilization, use of aspirin, and paracetamol	8
Ji	2009	Mixed	Mixed	NA	Obtain from register databases	Obtain from register databases	40	140425	No	7
Colak	2015	Mixed	Mixed	56	Self-reported	Obtain from register databases	9.4	94097	Age, sex, body mass index, familial pre-disposition for asthma, allergy, childhood asthma, hay fever, or eczema, use of asthma medication, occupational exposure to dust and/or fumes, daily exposure to passive smoking, physical activity in leisure-time, education, annual household income, and cumulative tobacco consumption	8
Huang 1	2015	Asian	Male	> 20	Obtain from register databases	Obtain from register databases	4	8002536	Lung diseases, low income, age, comorbidities, urbanization and geographic area	9
Huang 2	2015	Asian	Female	> 20	Obtain from register databases	Obtain from register databases	4	7216488	Lung diseases, low income, age, comorbidities, urbanization and geographic area	9
Fan	2016	Asian	Mixed	40–9	Physician-diagnosed	Chest radiography, physician-diagnosed, obtain from register databases	9	9295	Age, sex, education, smoking, arsenic level, radon level, prior comorbidities	8
Pirie	2016	Mixed	Female	56	Questionnaire	Obtain from register databases	14	634039	Age, region, deprivation quintile, height	7

NA, not available.

### Association of asthma and risk of lung cancer

**Figure 2:**
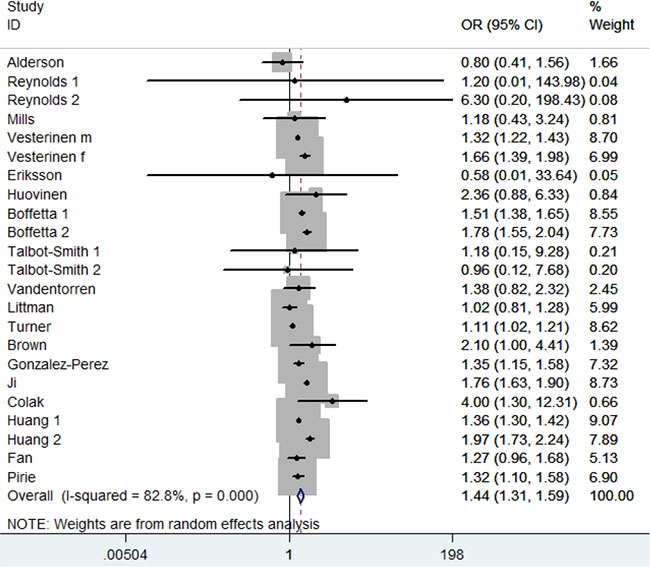
Meta-analysis of the association between asthma and lung cancer

As shown in Figure 2, asthma was significantly associated with the increased risk of lung cancer (odds ratio (OR) = 1.44; 95% confidence interval (CI) 1.31–1.59; *P* < 0.00001; *I*^2^ = 83%). Additionally, asthma patients without smoking also had the increased lung cancer risk (OR = 1.28; 95% CI 1.10–1.50; *P* = 0.002; *I*^2^ = 0%). In the subgroup analysis of race, both Caucasians and Asians with asthma showed the same results (OR = 1.53; 95% CI 1.37–1.72; *P* < 0.00001; *I*^2^ = 56%; OR = 1.52; 95% CI 1.15–2.01; *P* < 0.00001; *I*^2^ = 93%). In the stratified analysis by gender, both male and female patients with asthma showed the increased risk of lung cancer (OR = 1.38; 95% CI 1.31–1.46; *P* < 0.00001; *I*^2^ = 24%; OR = 1.68; 95% CI 1.45–1.95; *P* < 0.00001; *I*^2^ = 63%). However, asthma was not significantly associated with lung adenocarcinoma risk (OR = 1.01; 95% CI 0.69–1.50; *P* = 0.95; *I*^2^ = 45%). In the stratified analysis by asthma definition, significant associations were found between asthma and lung cancer in self-reported subgroup (OR = 1.23; 95% CI 1.03–1.48; *P* = 0.02; *I*^2^ = 53%), questionnaire subgroup (OR = 1.32; 95% CI 1.12–1.57; *P* = 0.001; *I*^2^ = 0%), and register databases subgroup (OR = 1.60; 95% CI 1.42–1.79; *P* < 0.00001; *I*^2^ = 91%). However, no significant association was observed in physician-diagnosed asthma subgroup (OR = 1.26; 95% CI 0.96–1.65; *P* = 0.10; *I*^2^ = 0%). The results were showed in Table 2.

**Table 2:** Results of this meta-analysis

	No. of study	OR (95% CI)	*P* Value for meta-analysis	*I^2^* (%)	*P* Value for subgroup analysis
Overall lung cancer risk	23	1.44 (1.31–1.59)	< 0.00001	83	
Non-smoker	4	1.28 (1.10–.50)	0.002	0	
Subgroup analysis					
Race					0.95
Caucasian	9	1.53 (1.37–1.72)	< 0.00001	56	
Asian	3	1.52 (1.15–2.01)	< 0.00001	93	
Gender					0.02
Male	6	1.38 (1.31–1.46)	< 0.00001	24	
Female	6	1.68 (1.45–1.95)	< 0.00001	63	
Lung adenocarcinoma	2	1.01 (0.69–1.50)	0.95	45	
Definition of asthma					0.05
Self-reported	9	1.23 (1.03–1.48)	0.02	53	
Questionnaire	3	1.32 (1.12–1.57)	0.001	0	
Physician-diagnosed	4	1.26 (0.96–1.65)	0.10	0	
Register databases	7	1.60 (1.42–1.79)	< 0.00001	91	

**Figure 3:**
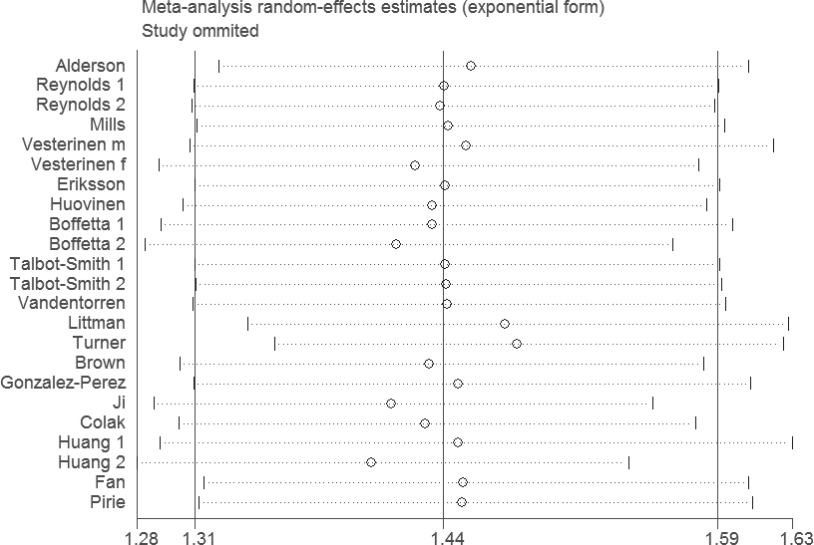
Sensitivity analysis of the association between asthma and lung cancer

In the sensitive analysis, similar data were observed after sequentially excluding each study (Figure 3). Furthermore, when the studies without adjustment were excluded, the result was still statistically significant (OR = 1.43, 95% CI 1.28–1.60, *P* < 0.00001; *I*^2^ = 80%). In addition, after excluding the studies without adjusting smoking and age, the result was not changed (OR = 1.29, 95% CI 1.12–1.48, *P* = 0.0004; *I*^2^ = 27%). The results were showed in Table 3.

**Table 3:** Results of sensitivity analysis

	No. of study	OR (95% CI)	*P* Value	*I^2^* (%)
Studies with adjustment	18	1.43 (1.28–1.60)	< 0.00001	80
Adjustment with smoking and age	12	1.29 (1.12–1.48)	0.0004	27

**Figure 4:**
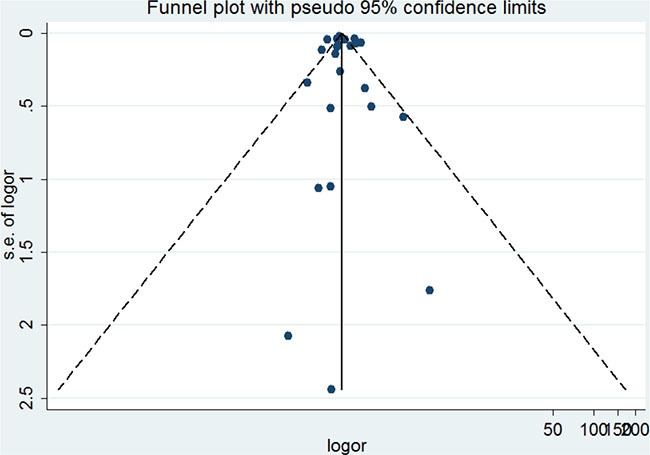
Funnel plot of the association between asthma and lung cancer

The publication bias of the included studies was assessed by the funnel plot and Egger's test. The funnel plot was symmetric (Figure 4). Egger's test showed no significant publication bias (*P* = 0.683).

## DISCUSSION

This present meta-analysis investigating the relationship between asthma and lung cancer risk. Eighteen studies with a total of 16375202 individuals were included in this meta-analysis. Prior asthma was significantly associated with lung cancer risk. In the subgroup analysis of race, both Caucasians and Asians with asthma showed the same results. In the stratified analysis by gender, both male and female patients with asthma showed the increased risk of lung cancer. Smoke habit was a recognized risk factor for lung cancer. However, asthma patients without smoking also had the increased lung cancer risk. These results suggested that asthma might be an independent risk factor for lung cancer. Lung cancer is classified small cell lung cancer and adenocarcinoma, squamous cell carcinoma, and large cell carcinoma [23]. Asthma was not significantly associated with lung adenocarcinoma risk in this meta-analysis. Future studies should be performed to assess the association between asthma and other pathological types of lung cancer. In the stratified analysis by asthma definition, significant associations were found in self-reported subgroup, questionnaire subgroup, and register databases subgroup. However, no significant association was observed in physician-diagnosed asthma subgroup. Only four studies were included in this subgroup. A positive association could therefore not be ruled out, because studies with small sample sizes may have had insufficient statistical power to detect any slight effect. To determine the stability of the result, we did sensitivity analysis. Removal of each study did not change the result, suggesting the reliability of our result. The adjusted ORs could be used to overcome some of the confounding within the observational studies. Thus, we did sensitivity analysis by excluding the studies without adjustment. The result was still statistically significant.

Asthma is a disease of chronic airway inflammation characterized by recurrent episodes of wheezing, dyspnea, chest tightness, and cough. Inflammatory cell types, such as T and B lymphocytes, mast cells, eosinophils, basophils, neutrophils and dendritic cells, as well as structural cell types including epithelial and mesenchymal cells involved in airway inflammation [24]. Inflammation also plays a pivotal role in the pathogenesis of lung cancer. Ballaz and Mulshine indicated that chronic inflammation contributes to the process of lung carcinogenesis [25]. Azad et al. suggested that chronic inflammation-induced production of reactive oxygen/nitrogen species in the lung may predispose individuals to lung cancer [26].

This meta-analysis was stable and reliable. First, sensitivity analyses revealed that the results were robust. Second, no significant publication bias was found in this meta-analysis. Third, the sample size of this meta-analysis was large enough. Several limitations should be noted. First, other races, such as African, were not included. Second, heterogeneity was high in this meta-analysis. Third, subgroup analysis based on study level variables could be due to the potential for ecological fallacy. Therefore, more studies are required to confirm the results of this meta-analysis.

In conclusion, this meta-analysis suggested that asthma might be significantly associated with lung cancer risk.

## MATERIALS AND METHODS

### Publication search

Two authors (YLQ and JL) searched Pubmed, Embase, Chinese National Knowledge Infrastructure (CNKI) and Wanfang databases (www.wanfangdata.com.cn). We also checked American Society of Clinical Oncology (ASCO) meeting abstracts to find grey literature (http://meetinglibrary.asco.org/abstracts). Last search was updated in 10 Nov, 2016. We searched the bibliographies of identified studies and narrative reviews for additional citations. No language and time restriction were imposed in this meta-analysis. The detailed search strategy is showed in the Supplementary Material.

### Study selection

Two authors (YLQ and JL) searched the titles and abstracts obtained from the initial electronic search for potentially relevant studies for full review. Two authors (YLQ and LXZ) then assessed the full text of the retrieved studies to determine whether the study met the inclusion criteria. Studies included in this meta-analysis should meet the following criteria: (1) study design: prospective cohort study, cross-sectional study, and longitudinal study; (2) population: individuals without lung cancer; (3) exposure: asthma or wheeze; (4) comparison: individuals without asthma or wheeze; (5) outcome: relative risk (RR), hazard ratio (HR), or OR with corresponding 95% CI of lung cancer risk in overall population and in non-smokers. If serial studies of the same population from the same group were reported, the largest study was included. Reviews, meta-analyses, letters, and editorial articles were all excluded. We resolved disagreement by consensus.

### Data extraction and qualitative assessment

Two investigators (YLQ and JL) extracted the data independently. The following data were collected from each study: first author's name, year of publication, ethnicity, gender, age, asthma definition, lung cancer definition, duration of follow-up, sample size, and adjustment. The Newcastle–Ottawa Scale (NOS) was used to assess the quality of included studies.

### Statistical analysis

ORs with 95% CIs were used to calculate the strength of the association between asthma and lung cancer risk. Random effects model was used in this meta-analysis. Heterogeneity among studies was examined with *I*^2^ statistic and Q statistic. Subgroup analysis was carried out by smoke status, ethnicity, gender, subtype of lung cancer, and definition of asthma. Relative influence of each study on the pooled estimate was assessed by omitting one study at a time for sensitivity analysis. Sensitivity analysis was also performed by excluding studies without adjustment, studies without adjustment of age and smoking. Funnel plot and Begg's test were employed to evaluate publication bias. All statistical tests were performed using the STATA 11.0 software (Stata Corporation, College Station, TX). A *P* value < 0.05 was considered statistically significant, except for tests of heterogeneity where a level of 0.10 was used. All tests were two-sided.

## SUPPLEMENTARY MATERIALS FIGURES AND TABLES



## References

[1] Hecht SS (1999). Tobacco smoke carcinogens and lung cancer. J Natl Cancer Inst.

[2] Sun S, Schiller JH, Gazdar AF (2007). Lung cancer in never smokers—a different disease. Nat Rev Cancer.

[3] Koppelman GH, Stine OC, Xu J, Howard TD, Zheng SL, Kauffman HF, Bleecker ER, Meyers DA, Postma DS (2002). Genome-wide search for atopy susceptibility genes in Dutch families with asthma. J Allergy Clin Immunol.

[4] Coussens LM, Werb Z (2002). Inflammation and cancer. Nature.

[5] Alderson M (1974). Mortality from malignant disease in patients with asthma. Lancet.

[6] Reynolds P, Kaplan GA (1987). Asthma and cancer. Am J Epidemiol.

[7] Mills PK, Beeson WL, Fraser GE, Phillips RL (1992). Allergy and cancer: organ site-specific results from the Adventist Health Study. Am J Epidemiol.

[8] Vesterinen E, Pukkala E, Timonen T, Aromaa A (1993). Cancer incidence among 78,000 asthmatic patients. Int J Epidemiol.

[9] Eriksson NE, Holmén A, Högstedt B, Mikoczy Z, Hagmar L (1995). A prospective study of cancer incidence in a cohort examined for allergy. Allergy.

[10] Huovinen E, Kaprio J, Vesterinen E, Koskenvuo M (1997). Mortality of adults with asthma: a prospective cohort study. Thorax.

[11] Boffetta P, Ye W, Boman G, Nyré n (2002). Lung cancer risk in a population-based cohort of patients hospitalized for asthma in Sweden. Eur Respir J.

[12] Talbot-Smith A, Fritschi L, Divitini ML, Mallon DF, Knuiman MW (2003). Allergy, atopy, and cancer: a prospective study of the 1981 Busselton cohort. Am J Epidemiol.

[13] Vandentorren S, Baldi I, I Annesi Maesano, Charpin D, Neukirch F, Filleul L, Cantagrel A, Tessier JF (2003). Long-term mortality among adults with or without asthma in the PAARC study. Eur Respir J.

[14] Littman AJ, Thornquist MD, White E, Jackson LA, Goodman GE, Vaughan TL (2004). Prior lung disease and risk of lung cancer in a large prospective study. Cancer Causes Control.

[15] Turner MC, Chen Y, Krewski D, Ghadirian P, Thun MJ, Calle EE (2005). Cancer mortality among US men and women with asthma and hay fever. Am J Epidemiol.

[16] Brown DW, Young KE, Anda RF, Felitti VJ, Giles WH (2006). Re: asthma and the risk of lung cancer. findings from the Adverse Childhood Experiences (ACE). Cancer Causes Control.

[17] González-Pérez A, Fernández-Vidaurre C, Rueda A, Rivero E, García Rodríguez LA (2006). Cancer incidence in a general population of asthma patients. Pharmacoepidemiol Drug Saf.

[18] Ji J, Shu X, Li X, Sundquist K, Sundquist J, Hemminki K (2009). Cancer risk in hospitalised asthma patients. Br J Cancer.

[19] Çolak Y, Afzal S, Nordestgaard BG, Lange P (2015). Characteristics and Prognosis of Never-Smokers and Smokers with Asthma in the Copenhagen General Population Study. A Prospective Cohort Study. Am J Respir Crit Care Med.

[20] Huang JY, Jian ZH, Nfor ON, Ku WY, Ko PC, Lung CC, Ho CC, Pan HH, Huang CY, Liang YC, Liaw YP (2015). The effects of pulmonary diseases on histologic types of lung cancer in both sexes: a population-based study in Taiwan. BMC Cancer.

[21] Fan Y, Jiang Y, Hu P, Chang R, Yao S, Wang B, Li X, Zhou Q, Qiao Y (2016). Modification of association between prior lung disease and lung cancer by inhaled arsenic: A prospective occupational-based cohort study in Yunnan, China. J Expo Sci Environ Epidemiol.

[22] Pirie K, Peto R, Green J, Reeves GK, V; Beral, Million Women Study Collaborators (2016). Lung cancer in never smokers in the UK Million Women Study. Int J Cancer.

[23] Travis WD (2011). Pathology of lung cancer. Clin Chest Med.

[24] Pelaia G, Vatrella A, Maselli R (2012). The potential of biologics for the treatment of asthma. Nat Rev Drug Discov.

[25] Ballaz S, Mulshine JL (2003). The potential contributions of chronic inflammation to lung carcinogenesis. Clin Lung Cancer.

[26] Azad N, Rojanasakul Y, Vallyathan V (2008). Inflammation and lung cancer: roles of reactive oxygen/nitrogen species. J Toxicol Environ Health B Crit Rev.

