# Correction: Asthma and the risk of lung cancer: a meta-analysis

**DOI:** 10.18632/oncotarget.19302

**Published:** 2017-07-17

**Authors:** Yan-Liang Qu, Jun Liu, Li-Xin Zhang, Chun-Min Wu, Ai-Jie Chu, Bao-Lei Wen, Chao Ma, Xu-yan Yan, Xin Zhang, De-Ming Wang, Xin Lv, Shu-Jian Hou

**Present:** The author's affiliations are listed incorrectly.

**Correct:** The proper affiliations are listed below.

Yan-Liang Qu1,*, Jun Liu2,*, Li-Xin Zhang1, Chun-Min Wu1, Ai-Jie Chu1, Bao-Lei Wen1, Chao Ma1, Xu-yan Yan1, Xin Zhang1, De-Ming Wang1, Xin Lv2, Shu-Jian Hou3

1 Department of Anesthesiology, No. 401 Hospital of PLA, Qingdao 266071, Shandong, China

2 Department of Anesthesiology, Shanghai Pulmonary Hospital, Tongji University School of Medicine, Shanghai, China

3 Department of Hand Surgery, No. 401 Hospital of PLA, Qingdao 266071, Shandong, China

Original article: Oncotarget. 2017; 8:11614-11620. https://doi.org/10.18632/oncotarget.14595

