# Supplementary material for: Asthma and the risk of lung cancer: a meta-analysis
Source: Oncotarget. 2017 Jan 11;8(7):11614–20. doi: 10.18632/oncotarget.14595 (PMC5355290; doi:10.18632/oncotarget.14595)
Supplement: Supplementary file 1 [file oncotarget-08-11614-s001.pdf]

Asthma and the risk of lung cancer: a meta-analysis

Supplementary Materials

SUPPLEMENTARY METHODS

Search Strategy

1. exp Asthma/
2. asthma$.mp.
3. Respiratory Sounds/
4. wheez$.mp.
5. Bronchial Spasm/
6. bronchospas$.mp.
7. (bronch$ adj3 spasm$).mp.
8. bronchoconstrict$.mp.
9. exp Bronchoconstriction/
10. (bronch$ adj3 constrict$).mp.
11. Bronchial Hyperreactivity/
12. Respiratory Hypersensitivity/
13. or/1-12
14. exp Lung Neoplasms/
15. (lung$ adj3 canc$).mp.
16. (lung$ adj3 carcinoma$).mp.
17. (lung$ adj3 tumo?r$).mp.
18. (lung$ adj3 neoplasm$).mp.
19. or/14-18
20. 13 and 19
